# Synthesis of Thermoresponsive Chitosan-*graft*-Poly(*N*-isopropylacrylamide) Hybrid Copolymer and Its Complexation with DNA

**DOI:** 10.3390/polym16101315

**Published:** 2024-05-08

**Authors:** Marius-Mihai Zaharia, Florin Bucatariu, Maria Karayianni, Elena-Daniela Lotos, Marcela Mihai, Stergios Pispas

**Affiliations:** 1 Petru Poni Institute of Macromolecular Chemistry, 41A Grigore Ghica Voda Alley, 700487 Iasi, Romania; zaharia.marius@icmpp.ro (M.-M.Z.); fbucatariu@icmpp.ro (F.B.); m.karayianni@icmpp.ro (M.K.); daniela.lotos@icmpp.ro (E.-D.L.); 2 Theoretical and Physical Chemistry Institute, National Hellenic Research Foundation, 48 Vassileos Constantinou Ave., 116 35 Athens, Greece

**Keywords:** chitosan, poly(*N*-isopropylacrylamide), RAFT polymerization, grafting, thermal response, DNA polyplexes, gene delivery systems

## Abstract

A hybrid synthetic-natural, thermoresponsive graft copolymer composed of poly(*N*-isopropyl acrylamide) (PNIPAM) side chains, prepared via RAFT polymerization, and a chitosan (Chit) polysaccharide backbone, was synthesized via radical addition-fragmentation reactions using the “grafting to” technique, in aqueous solution. ATR-FTIR, TGA, polyelectrolyte titrations and ^1^H NMR spectroscopy were employed in order to validate the Chit-*g*-PNIPAM copolymer chemical structure. Additionally, ^1^H NMR spectra and back conductometric titration were utilized to quantify the content of grafted PNIPAM side chains. The resulting graft copolymer contains dual functionality, namely both pH responsive free amino groups, with electrostatic complexation/coordination properties, and thermoresponsive PNIPAM side chains. Particle size measurements via dynamic light scattering (DLS) were used to study the thermoresponsive behavior of the Chit-*g*-PNIPAM copolymer. Thermal properties examined by TGA showed that, by the grafting modification with PNIPAM, the Chit structure became more thermally stable. The lower critical solution temperature (LCST) of the copolymer solution was determined by DLS measurements at 25–45 °C. Furthermore, dynamic and electrophoretic light scattering measurements demonstrated that the Chit-*g*-PNIPAM thermoresponsive copolymer is suitable of binding DNA molecules and forms nanosized polyplexes at different amino to phosphate groups ratios, with potential application as gene delivery systems.

## 1. Introduction

Gene therapy has gained popularity among scientists in recent decades as an alternative therapeutic strategy due to its numerous advantages over standard treatment methods [1,2,3]. The efficient transport of nucleic acids within the cells is heavily dependent on the deployment of a gene vector with the appropriate characteristics [4]. An effective gene carrier must be able to protect large DNA molecules (deoxyribonucleic acids) or small RNAs (ribonucleic acids) from both internal and external nuclease degradation while condensing them to sizes suitable for cellular absorption and intake [5]. Furthermore, in order to deliver nucleic acids to affected cells in a safe and effective manner, the carrier must be nonpathogenic, nonimmunogenic, and nontoxic. Nucleic acid delivery is critical because it enables the development of innovative drugs and therapeutic methodologies [6,7]. In contrast, nucleic acid transfer is connected with the insertion of genes into cells through vectors. Although viral carrier-based therapies are already approved, non-viral carriers are currently preferred due to their significant advantages and potential benefits, which include controlled chemical diversity, low cost, easier large-scale production, a higher payload capacity, improved safety profiles, and a lower immunogenic response [8,9].

Stimuli-responsive polymers are intriguing gene carriers because they may modify their interactions with DNA molecules in response to even small changes in their external environment [10]. RAFT (Reversible Addition Fragmentation Chain Transfer) polymerization is recognized as an adaptative and controllable radical polymerization process that has been widely used to produce various stimuli-responsive polymers and block copolymers by involving numerous functional monomers [11,12,13]. The RAFT polymerization technique offers several significant advantages, the most notable of which is the generation of molecular weight-controlled polymers with narrow molecular weight polydispersity, end group fidelity and complex macromolecular structures, which can be used in a variety of biomedical applications [14]. The wide range of functional RAFT agents and solvents accessible for polymerization, as well as the fact that RAFT polymerization necessitates less stringent experimental conditions and procedures than other polymerization approaches, are key advantages of this polymerization method [15]. Temperature-responsive poly(*N*-isopropylacrylamide) (PNIPAM), one of the widely known “smart” polymers, is soluble at ambient temperature in water and switch from coils to globules above its low critical solution temperature (LCST) located at 32 °C, due to the interactions between the hydrophobic isopropyl groups and the hydrophilic amide pendant groups [16,17]. The LCST value is near to physiological body temperature, making it an ideal polymer for gene transfer applications. At temperatures below LCST, the polymer is soluble in aqueous solution due to hydrogen bond formation between the amide side groups and water molecules, resulting in an extended or hydrated coil conformation [18]. As temperature rises, the polymer becomes less soluble and goes through a volume phase transition, folding into a globular form [19,20]. At this moment, intramolecular hydrogen bonds occur, and the polymer chains aggregate due to the increased hydrophobicity [21,22]. The grafting of PNIPAM chains onto natural polymer backbones (e.g., hyaluronic acid, chitosan, collagen, alginate, etc.) ensures temperature flexibility during gelling, biodegradation, and mechanical property control in hydrogels [18,23]. Due to growing environmental concerns, there is a lot of interest in the utilization of natural polysaccharides in many applications [24].

Chitosan (Chit) has many advantages when used as a drug delivery polymer. It is a biodegradable and biocompatible natural polymer with many benefits that include non-toxicity, bioactivity, affordability, and accessibility to modification by chemical addition to existing amino and hydroxyl groups [25,26]. Insulin, DNA, and vaccines have all been delivered using Chit and its derivatives as delivery vehicles in various forms, including film, hydrogel, complexes and microspheres [27,28]. High biocompatibility Chit can be chemically modified due to its amino (–NH_2_) and hydroxyl groups (–OH), which boost the polymer’s reactivity and make it pH-responsive by the NH_2_ protonation–deprotonation equilibrium. When Chit is deprotonated (pH > 6.3), it is water insoluble; when it is protonated (pH < 6.3), it gets water soluble [29]. Chit presents up as an exciting natural substrate for grafting by its unique joint of mechanical strength, reactivity, low price, and renewability. It also offers a high potential for chemical modification to suit innovative application areas [30,31]. Chitosan’s amino and hydroxyl groups can be modified using graft copolymerization to produce diverse macromolecular designs [32,33,34,35,36].

PNIPAM is an acrylamide synthetic polymer for grafting of Chit backbones [20,23,34,35,37]. Chit-*g*-PNIPAM copolymers have been identified as potentially thermoresponsive and pH-responsive materials for drug delivery [38,39,40] and tissue engineering [41,42]. Additionally, it has been shown that they improve the oral delivery of hydrophobic medications, such as naproxen, caffeine, and paclitaxel, and favors the mucosal delivery of hydrophobic pharmaceuticals [40]. Furthermore, cryogel scaffolds containing PNIPAM and Chit have been proposed for utilization in bioartificial liver devices and plasma purification. Also, nano and microgels made from Chit-*g*-PNIPAM have been proposed for oncological applications, such as bioactive drug delivery [43] and antibacterial applications [40,44]. Chit-*g*-PNIPAM copolymers are frequently employed in these applications, with a high proportion of NIPAM molecular units within the grafted Chit-*g*-PNIPAM structure [23,42,45,46]. Unfortunately, no information has been provided on the synthesis or thermoresponsive behavior of copolymers that cover a wide range of compositions, including those with low and high content of PNIPAM moieties, making it difficult to predict the parameters that determine the sharpness and intensity of the transition, which are critical for this kind of applications. Therefore, the current study aims to synthesize a hybrid synthetic-biological Chit-*g*-PNIPAM copolymer by applying the “grafting to” approach and using narrow distribution PNIPAM end-active chains produced by RAFT polymerization. The chemical structure of the obtained Chit-*g*-PNIPAM copolymer was validated by a gamut of techniques including ATR-FTIR, thermogravimetric analysis (TGA), polyelectrolyte titrations, back conductometric titrations and ^1^H NMR spectroscopy. Also, ^1^H NMR spectra were utilized to determine the amount of grafted PNIPAM side chains. Furthermore, light scattering measurements were employed to evaluate the aqueous solution thermoresponsive characteristics of the graft copolymer. Furthermore, the ability of the Chit-*g*-PNIPAM thermoresponsive copolymer to interact with DNA molecules was investigated. Specifically, a relatively short DNA macromolecule of approximately 113 bp was utilized to investigate the nucleic acid complexation ability of Chit-*g*-PNIPAM, the physicochemical properties of the formed copolymer/DNA complexes (polyplexes) at different N/P (amino to phosphate groups) ratios being evaluated through dynamic and electrophoretic light scattering measurements.

## 2. Materials and Methods

### 2.1. Materials

The water-soluble Chit (weight average molecular weight, MW = 162,000 g/mol; degree of deacetylation 88.2%), containing lactate and phosphates counterions, was acquired from Shandong AK Biotech Co., Ltd. (Jinan, China) and used without further modification. The PNIPAM was synthetized in our laboratory (MW = 3200 g/mol, PDI = 1.11), using a previously published protocol [47]. Potassium persulfate (KPS, 99%) and poly(ethylene sulfonic acid sodium salt) (PESNa), 4-cyano-4-[(dodecylsulfanylthiocarbonyl)sulfanyl] pentanoic acid as the chain transfer agent (CTA) and 2,2-azobis(isobutyronitrile) (AIBN) have been provided by Sigma-Aldrich (Sigma Chemical Co.; St. Louis, MO, USA). For stock solutions was used ultrapure water (conductivity 0.552 μS/cm) obtained by an EVOQUA Ultra Clear TPTWF (Evoqua Water Technologies LLC; Barsbüttel, Germany). DNA as sodium salt, derived from salmon sperm, with an average molecular weight MW = 75,000 g/mol (i.e., 50,000–100,000 g/mol) which corresponds to approximately 113 bp was received from Acros Organics (Geel, Belgium).

### 2.2. Synthesis of Chit-g-PNIPAM Copolymer

**Scheme 1:**
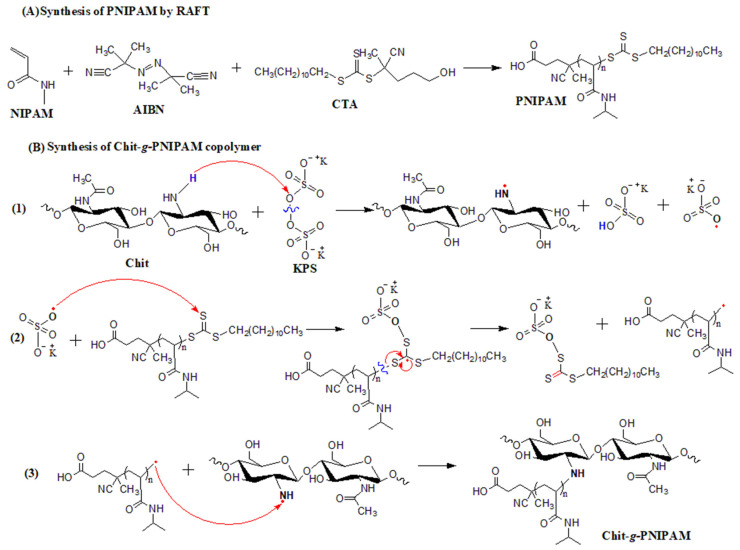
Schematical representation of (**A**) PNIPAM synthesis by RAFT polymerization and (**B**) proposed mechanism of the PNIPAM “grafting to” the Chit chains: (**1**) KPS extracts an H atom from the Chit amino group, generating a stable salt (potassium bisulfate) and Chit amino and bisulfate radicals; (**2**) the bisulfate radical attacks the PNIPAM chains at the trithiocarbonate active end group, generating a PNIPAM macroradical; (**3**) the PNIPAM macroradical reacts with Chit NH radical forming Chit-g-PNIPAM copolymer.

The reaction scheme for grafting PNIPAM to Chit chains is presented in Scheme 1. The “grafting to” approach is based on the anchoring of PNIPAM chains by their reactive chain ends to the polysaccharide via radical mediated coupling reaction, where the polymer reactive end moieties have been previously generated and prior to the final coupling toward copolymer synthesis.

PNIPAM homopolymer (Scheme 1A) was first synthesized by RAFT polymerization of NIPAM in a single step, in dioxane, at 70 °C for 6 h, using the pair of CTA and AIBN initiator in the polymerization reaction. The resultant homopolymer (PNIPAM) was separated and purified using double precipitation in excess n-hexane [47]. As illustrated in Scheme 1B, Chit-*g*-PNIPAM copolymer was synthesized by performing free-radical grafting, in inert atmosphere of nitrogen, of Chit (500 mg/4 mL purified water) with the active RAFT prepared PNIPAM homopolymer (300 mg/4 mL purified water), using KPS (6.4 mg/1 mL purified water) as the supplementary radicals producing agent for this synthesis step [23]. The reaction system was added in a round bottom flask fitted with an electrically operated magnetic stirrer at a temperature of 50 ± 1 °C, with constant stirring for 24 h. Before further analysis, the excess/unbonded PNIPAM was removed by subjecting the crude copolymer to a dialysis procedure (cellulose membrane with molecular weight cut-off, MWCO = 12,000–14,000 Da; Sigma Aldrich; St. Louis, MO, USA) during 5 days in ultrapure water at 25 °C with intermediate changes of the dialysis medium, followed by a freeze-drying procedure (freeze-dryer ALPHA 1–2 LD plus, Donau Lab; Kiev, Ukraine), during 72 h at −50 °C and 0.040 mbar.

### 2.3. Preparation of Copolymer/DNA Polyplexes

To investigate the complexation between Chit-*g*-PNIPAM and the DNA molecule different polyplex dispersions at varying mixing ratios between the two components were prepared as follows. Initial stock solutions of 0.1 mg/mL graft copolymer in 0.5wt.%. acetic acid (to facilitate the full protonation of the Chit amino groups) and 0.14 mg/mL DNA in water for injection (WFI) were prepared and left to equilibrate overnight. Subsequently, appropriate volumes of the DNA solution, that is 0.25, 0.5, 1 and 2 mL, were added under stirring to 1 mL of the Chit-*g*-PNIPAM solution. During this stage cloudy solutions were observed in some cases, indicating the formation of large complexes or aggregates. Finaly all solutions were diluted to the same final volume of 5 mL with addition of WFI, to have the same concentration of Chit-*g*-PNIPAM. The pH of the polyplex solutions is equal to that of WFI (due to the final dilution), which is about 6.5. The concentrations and mixing volumes of the initial stock solutions were chosen accordingly so as the estimated molar ratio of amino to phosphate groups, N/P, of the complexes is equal to 0.5, 1, 2 and 4. After equilibration the solution at N/P = 0.5 exhibited precipitation and the corresponding measurements were performed on the supernatant. Table 1 summarizes the polyplexes characteristics taking into account the final concentrations and N/P mixing ratios of the two components.

### 2.4. ATR-FTIR Analysis

The ATR-FTIR spectra were recorded by using an IR Tracer-100 FT-IR spectrometer (Shimadzu Corporation; Kyoto, Japan) equipped with a GladeATR module (PIKE Technologies; Madison, WI, USA). Samples were scanned in the 400–4000 cm^−1^ spectral region at 256 scans/spectrum with a resolution of 4 cm^−1^.

### 2.5. ^1^H NMR Spectroscopy

^1^H NMR spectroscopy was used to investigate the structure of Chit, PNIPAM and Chit-*g*-PNIPAM polymers. Spectra were collected at 25 °C using a 400 MHz Bruker Neo-1 equipment (Bruker; Rheinsteitten, Germany). To prepare the NMR sample solution, 50 mg of sample was dissolved in 800 µL of D_2_O while stirring at 60 °C. 2D NMR plots were created using the initial spectra. Advanced Chemistry Development Inc. (Toronto, ON, Canada) produced the ACD/Spectrus Processor software 2022.2.3, was applied to improve peak assignment, data processing, and comprehension. The integrals ratio of the signals at 2.07 ppm (related to the Chit) and at 1.16 ppm (related to PNIPAM side chains) were employed to estimate the molar percentages of Chit and PNIPAM in the graft copolymer after the purification step, as follows [48]:

PNIPAM(%) = (IP/n × 100)/((H/n) + (Ac:nHAc/DA)) (1)

Chit(%) = 100 − PNIPAM% (2)

where IP represent the isopropyl group integration peak area, n denotes the number of protons of the isopropyl group in NIPAM segment, Ac is the acetyl group integration peak area, nHAc represent the number of acetyl group protons (3), and DA the degree of acetylation of Chit (11.8%).

The degree of deacetylation (DD) of Chit was confirmed by ^1^H NMR using equation [49]:

DD(%) = (^1^HD/(^1^HD + ^1^HAc:3)) × 100 (3)

where ^1^HD and ^1^HAc represent the integrals of the peaks of the protons of deacetylated monomer units and of the three protons of *N*-acetyl group, respectively.

### 2.6. Thermogravimetric Analysis

The STA 49 F1 JUPITER thermogravimetric analyzer (Netzsch; Selb, Germany) was used to analyze Chit, PNIPAM, and the graft copolymer. The ceramic pan was loaded with approximately 5 mg of dried material and the analyses were performed in a nitrogen atmosphere (50 mL·min^−1^ flow) with a heating rate of 10 °C/min from 37 to 700 °C in order to assess the sample thermal stability and extract information regarding its composition. The experimental results were processed using the TGA equipment software (Netzsch Protens 4.2).

### 2.7. Polyelectrolyte Titration

The charge density of Chit, PNIPAM and Chit-*g*-PNIPAM were determined using a PCD 03 particle charge detector (Mütek GmbH; Neckartailfingen, Germany). For this, 1 mL of the 1 mg/mL solution of polymer was titrated using a strong polyanion i.e., poly(ethylene sulfonic acid sodium salt) (PESNa, 0.001 M), employing two methods (method 1—the initial solution was titrated with PESNa, followed by the HCl ionization of the residual functional groups and a further titration of the functional groups or, method 2—the ionization of all the functional groups in HCl, followed by the titration with PESNa).

### 2.8. Conductometric Analysis

The back conductometric titrations of Chit, PNIPAM and Chit-*g*-PNIPAM water solutions were carried out using a SevenExcellence pH/Cond meter S470-Std-K (Mettler-Toledo GmbH, Greifensee, Switzerland). The PNIPAM (10 mL, 1.2 mg/mL) solution conductivity containing excess of NaOH (20 mL, 1 mM) was measured during HCl (1 mM) titration. In the case of Chit and Chit-*g*-PNIPAM solutions (10 mL, 1.2 mg/mL), where HCl (10 mL, 10 mM) was in excess, the back conductometric titration has been carried out with NaOH (10 mM).

### 2.9. Particle Size Analysis

DLS measurements were used to assess particle size, scattered light intensity, size polydispersity index of graft copolymer, and the copolymer aggregation state as a function of temperature. A Zetasizer Nano ZS (Malvern Instruments, Malvern; Worcestershire, UK) with a 4 mW He-Ne Laser (633 nm incident laser wavelength) was used. The measurements were taken in ultrapure water solution at a scattering angle of 90° and, by means of a SOP Player, raising the temperature between 25 and 45 °C at 5 °C increments followed by cooling of the solutions back to 25 °C, with each measurement starting after a 10 min equilibration period (obtained values are the mean of three measurements).

In a similar manner, the polyplexes of Chit-*g*-PNIPAM/DNA were analyzed through DLS measurements utilizing an ALV/CGS-3 compact goniometer (ALVGmbH, Langen, Germany), connected with an multi tau digital correlator (ALV-5000/EPP), having a 632.8 nm He-Ne laser and an avalanche photodiode detector. The samples were introduced in 1 cm width standard cylindrical cells and analyzed at 90° angle and 25 °C, with each measurement being the average of 5 consecutive runs of 30 s. The obtained DLS measurements were evaluated by using the second order cumulant expansion or the CONTIN algorithms to calculate the hydrodynamic radius *R*_h_, using the Stokes-Einstein relationship.

### 2.10. Electrophoretic Light Scattering-Zeta Potential

Electrophoretic light scattering (ELS) measurements were performed at a 173° scattering angle and at room temperature, on a Zetasizer Nano-ZS (Malvern Panalytical Ltd., Malvern, UK) equipped with a He-Ne laser (λ = 633 nm) and an avalanche photodiode detector. The Smoluchowski approximation of the Henry equation was used for the calculation of zeta potential (*ζ*_P_) values.

## 3. Results

### 3.1. Characterization of Chit-g-PNIPAM Copolymer

The proposed reaction mechanism between the amino group of Chit and the end group of PNIPAM synthesized by RAFT polymerization using KPS as initiator are described in Scheme 1B. In the first reaction, the potassium persulfate, under the influence of temperature, can extract an H atom from the Chit amino group, generating in the system a stable salt (potassium bisulfate) and two radicals, Chit amino radical and bisulfate radical. The last one, more reactive than polymeric radical, could attack the PNIPAM chains at the trithiocarbonate active end group, generating a PNIPAM radical (reaction 2). This macroradical can react further with Chit radical forming Chit-*g*-PNIPAM graft-copolymer (reaction 3).

#### 3.1.1. ATR-FTIR Spectroscopy

**Figure 1:**
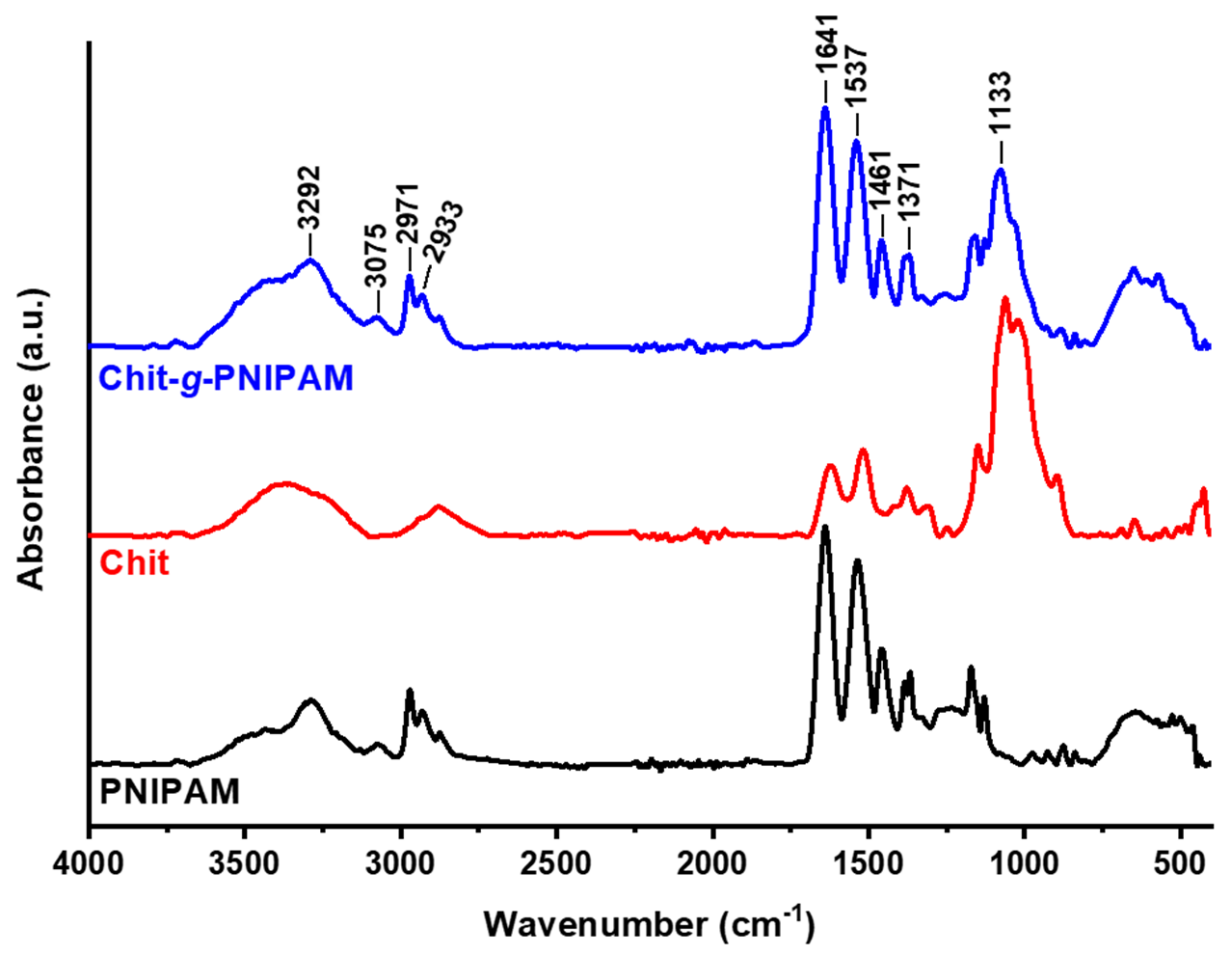
ATR-FTIR spectra of PNIPAM, Chit and Chit-*g*-PNIPAM copolymer.

The presence of PNIPAM and Chit was confirmed by ATR-FTIR analysis of Chit–*g*–PNIPAM copolymer (Figure 1).

The amine stretching N–H mode of PNIPAM was identified at 3292 cm^−1^ [50]. Additionally, the stretching vibration of carbonyl, as Amide I, was identified at 1641 cm^−1^ and the bending vibration of N–H (Amide II) at 1537 cm^−1^ [23]. The PNIPAM isopropyl group shows peaks at 2971 cm^−1^ and 2933 cm^−1^ corresponding to the –CH group (symmetric and asymmetric), as well as a couple of peaks at 1371 cm^−1^ and 1461 cm^−1^ attributed to the –CH_3_ group. The band at 1133 cm^−1^ was correlated to C–O stretching in Chit, indicating the copolymer structure. The standard bands of N–acetyl groups in the Chit backbone at about 1618 cm^−1^ (C=O, Amide I) and 1325 cm^−1^ (C–N, amide III), were obscured by the high concentration of PNIPAM, which displayed peaks at the approximatively same wavenumbers. The graft copolymer exhibited both characteristic patterns for PNIPAM and Chit. Also, the synthesized graft-copolymer did not contain sulfur atoms in the structures proved by its ATR-FTIR spectrum (i.e., the disappearance of peaks at 860 and 610 cm^−1^ wavenumbers from Chit-g-PNIPAM spectrum).

#### 3.1.2. ^1^H NMR Spectroscopy

^1^H NMR spectroscopy was employed to confirm the structure of the hybrid graft copolymer.

**Figure 2:**
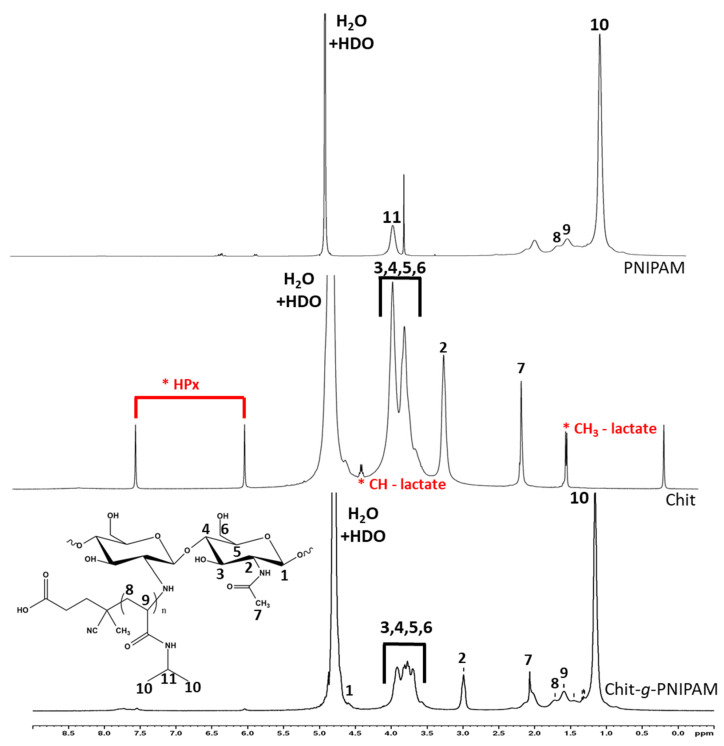
Validation of Chit-*g*-PNIPAM structure via ^1^H NMR. The spectra of PNIPAM, Chit and Chit-*g*-PNIPAM are shown in comparison.

Figure 2 depicts the proton NMR spectra of Chit, PNIPAM, and Chit-*g*-PNIPAM. The graft copolymer spectrum (Chit-*g*-PNIPAM) shows signals at 1.16 ppm (NMR peak 10), 1.72 ppm (peak 8), and 1.46 ppm (peak 9) which correspond to the methyl (CH_3_), ethyl (CH_2_), and vinyl (CH) protons, respectively, in the PNIPAM structure [51]. Also, the signal at 2.07 ppm (peak 7) belongs to CH_3_ protons in the acetyl group, and the signal at 4.80 ppm is from residual water in the D_2_O solvent [52]. Furthermore, the sharp peak at 4.46 ppm (peak 1) corresponds to H_1_ and the peak at 3.00 ppm (peak 2) relates to H_2_ of the Chit pyranose repeating unit [45]. Additionally, the peaks ranging from 3.51 to 3.92 ppm (NMR peaks 3, 4, 5, 6) are ascribed to H_3_-H_6_ of the pyranose ring in Chit [46]. The two reactive groups in the Chit backbone involved in the grafting process are the NH_2_ groups located in the deacetylated units (C_2_) and the OH groups at C_3_ and C_6_ [53]. The majority of grafting reactions supposedly happened at C_2_ due to its increased reactivity as compared to OH groups of Chit [54]. In keeping with prior studies [55], the absence of ^1^H NMR peaks associated with C_2_ from in the pyranose ring of Chit *D*-glucosamine monomeric units suggested that this site was employed as reactive site for grafting PNIPAM chains in the Chit-*g*-PNIPAM synthesis.

The content of PNIPAM and Chit in the Chit-*g*-PNIPAM was also calculated by ^1^H NMR spectroscopy. DD of Chit, calculated from ^1^H NMR using equation (3) is 88.06%, in accordance with the results obtained in a previously study when Chit DD was determined by ATR-FTIR (DD = 87.92%) and XRD (DD = 88.3%) [56], with very small differences between the methods and in accordance with the DD provided by the producer (DD = 88.2%). The weight percentages of PNIPAM and Chit in the graft copolymer were estimated by comparing the integrals of the signal at 2.07 ppm corresponding to the acetyl group of Chit to those of the signal at 1.16 ppm related to the isopropyl group of PNIPAM. Using Equations (1) and (2) it was obtained 19.9% PNIPAM and 80.1% Chit molar ratio in Chit-*g*-PNIPAM. From theoretical calculations, we assumed 975 monomeric units for Chit (860 deacetylated monomeric units and 114 acetylated monomeric units) and 25 monomeric units for PNIPAM. According to the ^1^H NMR calculations (19.9% PNIPAM) there are 356 monomeric units of NIPAM per Chit-*g*-PNIPAM chain. Since PNIPAM chain had 25 monomer units there are about 14 PNIPAM chains per grafted Chit; giving a grafting density of about one PNIPAM chain at every 70 Chit monomeric units, and thus the molecular weight of Chit-*g*-PNIPAM is approximatively 206,800 g/mol.

#### 3.1.3. Thermogravimetric Analysis

**Figure 3:**
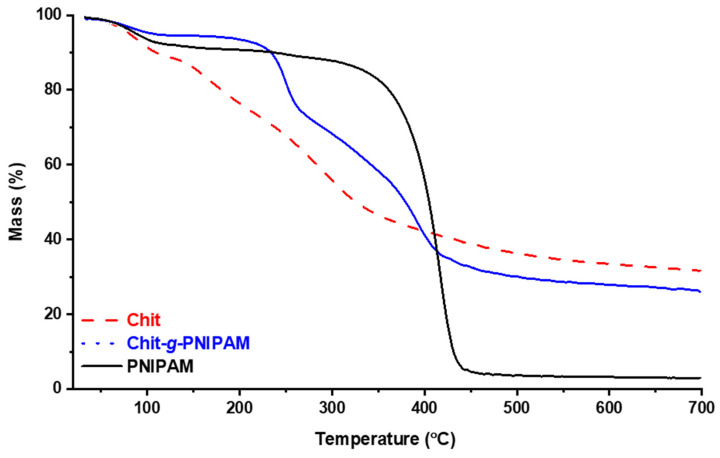
TGA curves of Chit, Chit-*g*-PNIPAM and PNIPAM polymers.

The TGA curves of Chit, PNIPAM, and obtained Chit-*g*-PNIPAM were registered, and their thermal behavior was compared (Figure 3).

Chit and PNIPAM showed different thermal degradation course, PNIPAM showing greater thermal stability until its complete decomposition at 450 °C. The TGA curve of Chit revealed two decomposition steps (Figure 3, dash line). The first happened at 38–96 °C due to water loss [57], with a weight loss of roughly 9.58%. The major decomposition began at 150 °C with a weight loss of about 44% and was caused by pyranose rings dehydration, then depolymerization, followed by disintegration of Chit backbone [55]. The degradation of PNIPAM was limited also to two steps (Figure 3, straight line), the first, which was detected at roughly 93 °C and included a mass loss of 8%, might be due to the water evaporation and the second one at 350–450 °C, PNIPAM being completely degraded in this temperature range. In the meantime, the three-step breakdown process was typical of the produced graft copolymer, Chit-*g*-PNIPAM (Figure 3, dot line). The first, at around 95 °C and accompanied by a mass loss of 4%, assigned to the evaporation of water found on the polymeric components retained by physical bonds (as hydrogen ones) to the polar groups of PNIPAM and Chit. The decomposition of the Chit backbone is responsible for the second considerable weight loss of the produced copolymer, around 200–300 °C (26% mass loss). The third step starting at 310–410 °C with 35% mass loss, could be caused by the decomposition of the acrylamide groups from PNIPAM. The residual mass (25.57%), remained at 700 °C, was assigned to the Chit component in the graft copolymer, and the mass percentage of Chit component correspond to the Chit used during grafting with PNIPAM. The degradation temperature/stability for Chit in graft copolymer decreased from 250 to 210 °C, due to PNIPAM impact on the crystalline phase of Chit [58]. Fortunately, these thermal modifications occurred at temperatures which are irrelevant for possible medically applications, which never could be met in vivo.

#### 3.1.4. Polyelectrolyte Titrations

The charge density of the solid polymer samples, expressed as meq (charges)/g sample, was calculated based on polyelectrolyte titration experiments and the results are presented in Table 2. By calculating the charge density of Chit, PNIPAM and Chit-*g*-PNIPAM polymers, it can be observed that PNIPAM can be ionized at basic pH and its carboxylic functional end group is completely ionized in aqueous solution at basic pH, as expected for a weak polyelectrolyte. On the other hand, we could observe that in the case of Chit, more than 30% of the amine functional groups are uncharged in aqueous solution (neutral pH). The addition of a small amount of HCl results in a protonation of a substantial amount of amine groups. Based on the performed polyelectrolyte titrations, by comparing the charge density and the percent of initial components (Chit and PNIPAM) with those of Chit-*g*-PNIPAM copolymer, it can be noticed that the later has a higher number of functional groups which can be also ionized in aqueous solution at different pHs, proving indirectly the grafting reaction of PNIPAM to Chit backbone.

#### 3.1.5. Back Conductometric Titrations

**Figure 4:**
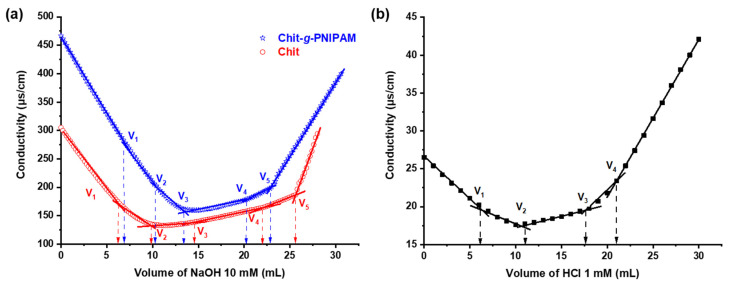
Back conductometric titration curves of (**a**) Chit and Chit-*g*-PNIPAM, and (**b**) PNIPAM solutions.

The mean volumes (V_1_, V_2_, and V_3_) of NaOH (10 mM) (Figure 4a) and (V_1_ and V_2_) of HCl (1 mM) (Figure 4b) were used to identify the equivalence points which correspond to each functional group capable to generate ions with different mobility in solution.

The acid-base functional groups of the Chit, PNIPAM and Chit-*g*-PNIPAM are mainly amino, carboxy and trithiocarbonate groups which can interact with NaOH and HCl in aqueous solution. Due to the decrease or increase of the hydronium and hydroxyl ions mobility different inflection points on the conductometric curves can be identified for each polymer. Using these inflection points in conductivity of aqueous solutions, it was possible to quantify each functional groups present on polymeric chains (Figure 4). Thus, the conductometric back-titration curve of the Chit aqueous solution presents five equivalent points. The first equivalent point located at V_1_ = 5 mL was assigned to the HCl excess, which remains in solution after the consumption of all basic functional groups present initially in solution. Chit backbone was acquired as a soluble Chit, that means the native sample contains lactate and phosphates, confirmed by the ^1^H NMR spectra (Figure 2). Therefore, the difference between the initial added volume (10 mL, 10 mM HCl) and V_1_ (titrated excess) has been attributed to the amino groups of Chit, while V_2_ − V_1_ to the lactic acid (K_a_ = 8.3 × 10^−4^) and H_3_PO_4_ (K_a1_ = 7.1 × 10^−3^), formed after the formation of -NH_3_^+^Cl^−^ (Figure 4a). The degree of hydrolysis (*x*) of Chit has been determined from the following equation:

[*x* × 272.17 + (1 − *x*) × 221.21] = m/[(V_HCl_ − V_1_ × f) × c] (4)

where: 272.17 is the average molar mass (g/mol) of the ammonium lactate or phosphate Chit structural unit; 221.21 is the molar mass (g/mol) of the acetylated structural units of Chit; V_HCl_ is the volume (L) of the initial added HCl with the concentration c (10 mM); f is the solution factor of the titrant (NaOH, 10 mM); V_1_ is the titrant volume (L) used to neutralize the HCl excess; m is the mass of the Chit sample used for back-titration (mg).

Therefore, from the first inflection point (V_1_) a degree of hydrolysis of 0.88 (or 88% amino groups) was calculated, which was further confirmed by ^1^H NMR measurements for soluble Chit. Also, based on the same volume V_1_, the charge density of soluble Chit was calculated, which was 3.78 meq/g Chit salt. From fourth (V_4_) and third (V_3_) equivalent points it is possible to calculate the charge density (5.5 meq/g Chit base) and the moles number of –NH_3_^+^Cl^−^ groups with hydrolysis constant k_H_ = 5.65 × 10^−10^. From the calculations nearly the same value for hydrolysis degree (86%) of Chit was found. The difference V_5_ − V_4_ corresponds to the titration of Na_2_HPO_4_ (K_a3_ = 4.6 × 10^−13^) which represents nearly half from the total number of amino groups (or ammonium groups).

Using the same conductometric back-titrations it is possible to determine the numeric molecular weight of the PNIPAM obtained by RAFT polymerization. Assuming that each polymeric chain has one carboxylic unit at the end it was possible to determine the carboxylic content of a certain mass of PNIPAM sample. From the first inflection point, V_1_ (Figure 4b) the total amount of acidic groups of the PNIPAM chains (carboxylic and trithiocarbonate groups) was calculated. Taking into account that each chain contains one carboxylic and one trithiocarbonate group, the number of functional groups determined from conductivity measurements must be divided by two to obtain the number of chains. Therefore, after titration of 12 mg PNIPAM with a solution of NaOH in excess (1 mM, factor 0.88) it was obtained approximately 9 μmoles of functional groups, resulting to a molar mass of 2700 g/mol for the RAFT synthesized PNIPAM, this value being close to the molar mass obtained by SEC. The second inflection point (V_2_) corresponded to the end of titration of the trithiocarbonate sodium salt functional group because the trithiocarbonate group could act like a Lewis acid in the reaction with HCl. The number of calculated functional groups being in this case equal to 4.5 μmoles, half from the total amount of functional groups which can be found in 12 mg PNIPAM. Further in the conductivity curve the third equivalence point can be obtained, V_3_, which corresponds to the titration of sodium carboxylate groups. In this case the calculated amount has been 5 μmoles, close to the trithiocarbonate amount. The final equivalent point, V_4_, indicated, also, the titration of trithiocarbonate groups, but in this case this chain end-group behaved like a Brönsted base in reaction with the HCl. Using the conductivity measurements of an aqueous PNIPAM solution, it was possible to calculate the functional groups content and, therefore, the molecular mass of the synthesized PNIPAM polymer. Using an aqueous solution of 12 mg (1 mg/mL) of graft-copolymer Chit-*g*-PNIPAM in the conductivity measurements, after the addition of an excess of HCl (10 mL, 10 mM), the positive charge mass density was calculated. Using the first inflection point, V_1_ (Figure 4a) and the initial added volume of HCl, it was found an approximate value of 3.5 meq (+)/g for the graft copolymer. Moreover, this charge density corresponds with to the total number of amino (primary and secondary) groups of the graft copolymer. During the RAFT polymerization, the dodecyltritiocarbonic acid was formed, this acid formed ammonium dodecyltritiocarbonate with primary and secondary amino groups of the grafted Chit. Therefore, in the reaction with HCl this ammonium salt liberates dodecyltritiocarbonic acid, a weaker acid than HCl, and ammonium chloride. All these acido-basic species could be identified in the back-conductometric curve of the Chit-*g*-PNIPAM graft copolymer and using the titrant volume corresponding to each inflexion point, it is possible to quantify the amount of each type of functional groups. In this regard, V_2_ − V_1_ and V_3_ − V_2_ have been attributed to the dodecyltritiocarbonic acid and PNIPAM end-carboxyl groups, respectively. These two groups represent the acidic groups present in the aqueous system, together with Chit primary and secondary ammonium chloride, determined from the differences V_4_ − V_3_ and V_5_ − V_4_. The number of carboxylic groups is equal to the number of secondary ammonium chloride groups and trithiocarbonic acid counter ion. The calculated molar ratio from conductometric curve confirmed this fact [(V_5_ − V_4_):(V_4_ − V_3_):(V_3_ − V_2_) = 1]. Also, from the molar ratio between secondary amine groups (titrated as ammonium chloride groups) of the graft copolymer and the total number of Chit-*g*-PNIPAM amine groups it was possible to estimate the degree of grafting {(V_5_ − V_4_)/[(V_5_ − V_4_) + (V_4_ − V_3_)] × 100 = 21.62%}. This calculated value from conductometric titrations is in accordance with the value determined from ^1^H NMR spectrum of Chit-*g*-PNIPAM as discussed above.

### 3.2. Aggregation State of Chit-g-PNIPAM on Temperature Changes

**Figure 5:**
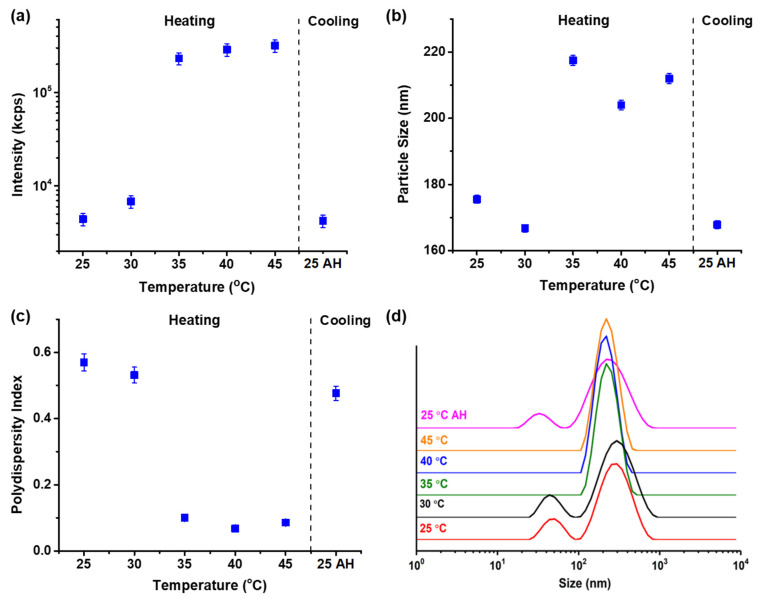
The influence of temperature on the: (**a**) scattered light intensity, (**b**) particle size, (**c**) polydispersity index and (**d**) size distribution of Chit-*g*-PNIPAM (polymer concentration: 1.2 mg/mL, pH 6.5, number of experiment replications = 3).

For an enhanced comprehension of the self-assembly of the graft copolymer in aqueous solution, the Chit-*g-*PNIPAM particle diameter, size polydispersity index, scattered light intensity and their stability on temperature variation were measured using DLS. The influence of the heating/cooling process (in the temperature range 25–45 °C) on all indicated parameters is shown in Figure 5.

The temperature range was established depending on the PNIPAM properties, which has an LCST around 32 °C. From Figure 5a, increasing the temperature impacts the mass of the Chit-*g*-PNIPAM aggregates as indicated by the increase of the scattered intensity (drastically increased after LCST of PNIPAM). At low temperatures (25 °C), because PNIPAM contains both groups with hydrophilic (carboxyl end group) and hydrophobic (isopropyl and backbone carbons) character, water molecules could organize as cage-like forms to enclose the PNIPAM hydrophobic parts. As temperature rises (after LCST), the “water cages” are destroyed and the hydrophobic groups are exposed, resulting in the production of hydrophobic aggregates of PNIPAM. The size of the Chit-*g*-PNIPAM did not vary significantly with temperature, suggesting that the already formed particles at low temperature combine with each other as temperature rises changing only their density and not their overall size upon temperature increase (Figure 5b). This goes in parallel with an increase in aggregate mass as scattered intensity increase indicates. This process can be assigned to the generation of more compact structures by the PNIPAM chains dehydration, based on both the hydrophobic interactions between the hydrophobic parts of Chit/PNIPAM and H-bonding between PNIPAM amides and Chit hydroxyls. In DLS, PDI (size polydispersity index) refers to the intensity size distribution spread over different size entities. Thus, polymers/aggregates having low PDI would present tight size distribution range [59]. Figure 5c shows that PDI values are above 0.2 at temperatures below the cloud point and drop under 0.2 at higher temperatures (after LCST), suggesting a shift from loose structure to more ordered/dense aggregates of Chit-*g*-PNIPAM [60]. The equivalent size distributions for Chit-*g*-PNIPAM copolymer (Figure 5d) demonstrate that above 35 °C, where PNIPAM chains possess enhanced hydrophobicity upon reaching the LCST, show only one population. Hydrogen-bonding between the graft macromolecules and water are diminished above the LCST. Therefore, the water clusters separate, allowing the hydrophobic isopropyl groups to become more closely associated. This causes the change of the configuration of the polymer chain from a relaxed curled conformation to a tighter globule. As a result of the hydrophobic (PNIPAM/PNIPAM and PNIPAM/Chit) and H-bonding (PNIPAM/Chit) association nano-phase separation occurs [61,62]. Nonetheless, after the cooling of Chit-*g*-PNIPAM solutions, the reversibility of the aggregation/solubilization process has been demonstrated, as can be seen in Figure 5 (last point in the plots). The thermoresponsive characteristics of the Chit-*g*-PNIPAM macromolecules, such as LCST and particle shrinking, were impacted by the dense PNIPAM core, which is more biocompatible than the PNIPAM homopolymer due to the Chit-conjugated shell structure. As a result, the thermoresponsive properties of Chit-*g*-PNIPAM graft copolymer synthesized make it potentially valuable for the construction of new forms of intelligent medicinal nanocarriers, more compatible with cells for to subsequent release of bioactive compounds (as a “*smart*” gene delivery system for example).

### 3.3. Complexation of Chit-g-PNIPAM Graft Copolymer with DNA

**Figure 6:**
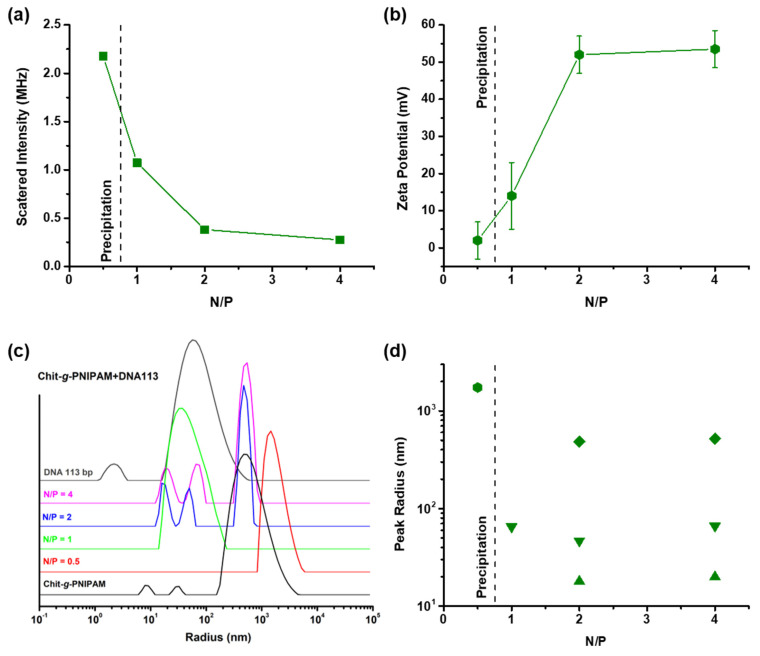
DLS and ELS results for the Chit-*g*-PNIPAM/DNA complexes were (**a**) the scattered intensity values, (**b**) the zeta potential values, (**c**) the size distributions of the hydrodynamic radii, *R*_h_, and (**d**) the *R*_h_ values of the peaks of the size distributions (the different symbols: triangle, inverted triangle, equilateral triangle, and diamond correspond to the different peaks according to size), depending on the N/P ratio.

As mentioned above, the Chit-*g*-PNIPAM copolymer can be potentially utilized for the successful carriage of nucleic acids, since for this type of applications it is important to have a polymeric carrier that offers essential advantages like stability, protection from the degradation of nuclease, ability to aim at specific cells and stimulate cellular entry [63]. Along these lines, the capacity of the PNIPAM-grafted Chit to interact electrostatically with a model DNA molecule, having a length of 113 bp, was examined by preparing electrostatic complexes (polyplex solutions) at different N/P (amino to phosphate groups) mixing ratios and investigating their solution properties. Figure 6 presents the results obtained by DLS and ELS on the scattered intensity, the zeta potential, the size distribution functions and the corresponding peak sizes, depending on the N/P ratio. Worth to mention that the solution at N/P = 0.5 exhibited precipitation and the corresponding measurements were performed on the supernatant.

As it can be seen, the mass of the complexes as evidenced by the corresponding scattered intensity values (Figure 6a) is especially high at low N/P ratios, thus providing proof of the complexation and even indicating the strong electrostatic interaction between the two macromolecular components. In parallel, the zeta potential values (Figure 6b) that express the effective charge of the complexes are significantly lower than that of pure Chit-*g*-PNIPAM (measured at +57 ± 4 mV), apparently as a result of the occurring charge neutralization due to electrostatic interactions and the shielding effects of grafted PNIPAM chains. This strong interaction is further supported by the observed precipitation at N/P = 0.5, which is most probably associated with the formation of very large/massive complexes or aggregates that are no longer colloidally stable, also evidenced by the corresponding effective charge of the particles which is close to zero. Even so, in the supernatant part of the solution large aggregates with high mass are still present as demonstrated by the corresponding size (Figure 6c,d) and intensity (Figure 6a) values. At N/P = 1 the formed complexes are characterized by rather high mass and quite smaller size (note that only one peak at about 70 nm is discerned in the size distribution), which can be attributed to structures with increased compactness and/or density. It seems that as Chit-*g*-PNIPAM copolymer interacts with the DNA chains the solubility of the formed complex is reduced mostly due to charge neutralization, thus leading to secondary aggregation and formation of large/dense aggregates. Moreover, it is also possible that the DNA chains act as bridging/binding agents between different copolymer chains further promoting aggregation. As the N/P ratio increases (N/P = 2 and 4), or equivalently the Chit-*g*-PNIPAM copolymer is in excess, the scattered intensity values become lower and are closer to that of the pure copolymer (~0.1 MHz, data not shown), suggesting that the complexes structures and properties are mostly dictated by those of the free copolymer. This is also the case for the zeta potential values that increase markedly and almost reach that of pure Chit-*g*-PNIPAM (about +57 mV, as mentioned above), as well as the sizes of the different peaks discerned in the size distribution functions which are similar to the ones observed for the copolymer. The observation of different scattering populations in the stock solution of Chit-*g*-PNIPAM suggests that the graft copolymer exhibits some degree of self-assembly, forming multi-chain aggregates. Respectively, in the solutions of the complexes where more than one peaks are observed (N/P = 2 and 4) the different populations apparently indicate the formation of complexes/aggregates comprising of different number of copolymer and DNA chains. Overall, the Chit-*g*-PNIPAM/DNA complexes structure and their solution properties are strongly influenced by the two components mixing ratio, as well as the initial conformation of the graft copolymer.

### 3.4. Thermal Response of the Chit-g-PNIPAM and DNA Complexes

**Figure 7:**
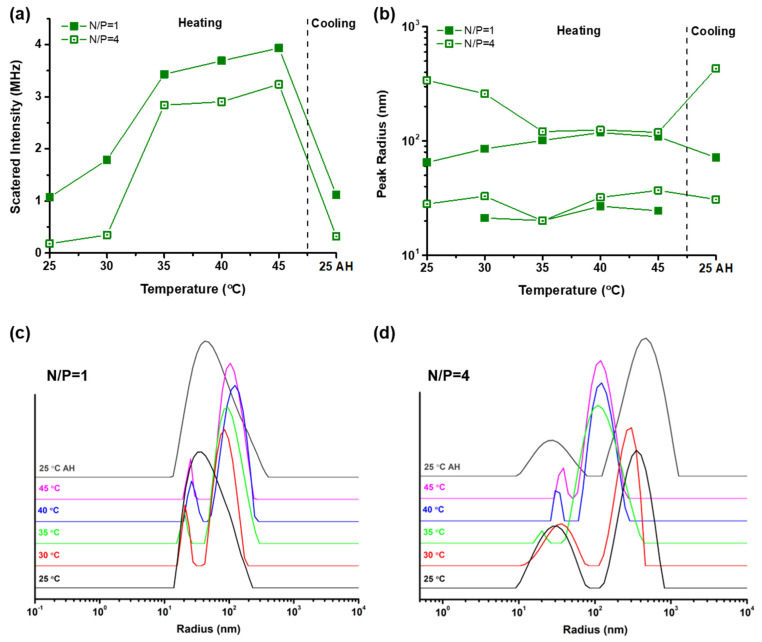
DLS results for representative Chit-*g*-PNIPAM and DNA complexes at N/P = 1 and 4 were (**a**) the scattered intensity values, (**b**) the *R*_h_ values of the peaks of the corresponding size distributions, and (**c**,**d**) the size distributions of the hydrodynamic radii, *R*_h_, are shown as a function of temperature.

The thermoresponsive character of the synthesized Chit-*g*-PNIPAM copolymer is expected to give additional functionalities to the formed copolymer/DNA polyplexes. For this reason, the thermal response of representative Chit-*g*-PNIPAM/DNA complexes at N/P = 1 and 4 was investigated by performing DLS measurements at different temperatures, that is from 25 to 45 °C with a 5 °C step, Figure 7 showing the corresponding results. A similar abrupt rise in scattered intensity is found above 30 °C for both complexes, in analogy to the one observed for the Chit-*g*-PNIPAM copolymer (Figure 5a). This change is attributed to the increase of the hydrophobicity of the PNIPAM grafts on Chit that further induces aggregation of the copolymer/DNA complexes. A rather interesting point here is that the observed intensity increase is greater in absolute value at higher N/P values, or in other words for the complex for which the Chit-*g*-PNIPAM copolymer is in excess. Since the effect of temperature on the complexes size is important, it seems that at N/P = 1 there is a slight increase of the corresponding size, evidenced by the distinction of two separate peaks (Figure 7c) with the position of the second gradually shifting to slightly higher *R*_h_ values, a fact that most likely signifies some degree of secondary aggregation. On the other hand, for the complex with N/P = 4 the size of the larger scattering population in solution decreases significantly above 30 °C, indicating the formation of more compact structures (i.e., shrinking) owning to the increase of the hydrophobicity. Once again, this behavior is closer to the one exhibited by the pure Chit-*g*-PNIPAM, which is fairly reasonable since the copolymer is in excess now. The detected differences in the temperature response of the two Chit-*g*-PNIPAM/DNA polyplexes at different mixing ratios highlight the significance of the predominant thermoresponsive character of the copolymer Chit-*g*-PNIPAM. For low N/P ratios the number of DNA chains interacting with each Chit-*g*-PNIPAM chain is higher, thus somewhat shielding/hindering the potential PNIPAM interchain aggregation or phase transition occurring above their respective LCST. As the N/P ratio becomes higher and there is a surplus of copolymer, the temperature-responsive nature of PNIPAM dictates to a large degree the complexes behavior. Nevertheless, in both cases the transitions are fully reversible upon cooling the solutions back to room temperature, demonstrating the ability of the system to return to its initial state.

**Figure 8:**
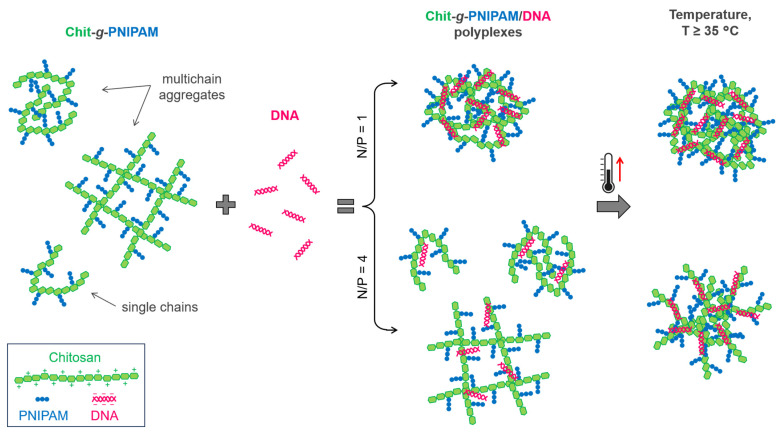
Schematic representation of the complexation process between the Chit-*g*-PNIPAM copolymer and the DNA chains, at two N/P ratios and their response to the increase of temperature (components are not drawn exactly in scale).

In an attempt to provide a more comprehensive illustration of the systems under study, a schematic representation of the complexation process between the Chit-*g*-PNIPAM copolymer and the DNA chains, showing the structure of the formed polyplexes at representative N/P ratios and their response to the increase of temperature is given in Figure 8.

## 4. Conclusions

A thermoresponsive Chit-*g*-PNIPAM copolymer was successfully obtained by the “grafting to” methodology based on the covalent grafting of PNIPAM chains, by RAFT polymerization, to Chit. The formation of Chit-*g*-PNIPAM copolymer was demonstrated by TGA, ATR- FTIR and ^1^H NMR spectroscopy, polyelectrolyte and back conductometric titrations. Furthermore, the grafting efficiency was quantified as 19.9% by ^1^H NMR and 21.62% by back conductometric titration. The Chit-*g*-PNIPAM hybrid copolymer showed thermoresponsive self-assembly in aqueous solutions as exemplified by light scattering measurements in relation to temperature (temperature range 25–45 °C). Apparently, a dense PNIPAM core is formed at higher temperatures shielded by a Chit structure, while continuous particle shrinkage takes place. Dynamic and electrophoretic light scattering measurements demonstrate that Chit-*g*-PNIPAM interacts electrostatically with a short DNA molecule (113 bp length), producing polyplexes of different structural characteristics and thermoresponsive behavior depending on the N/P mixing ratios. It was established that the initial conformation of the graft copolymer and the mixing ratio of the two components have a substantial influence on the composition and in the characteristics of the resultant solution Chit-*g*-PNIPAM/DNA complexes. Consequently, the obtained Chit-*g*-PNIPAM graft copolymer with its thermosensitive properties could be advantageous in developing novel intelligent nanocarriers that are more compatible to cells for the transport and delivery of nucleic acids.

## Figures and Tables

**Table 1:** The name of the sample, the concentration of Chit-*g*-PNIPAM and DNA in the solution, the total concentration and the estimated amino to phosphate groups molar ratio, N/P, for the Chit-*g*-PNIPAM/DNA polyplexes in WFI.

Sample Name	C_Chit-*g*-PNIPAM_ (mg/mL)	C_DNA_ (mg/mL)	C_Total_ (mg/mL)	N/P
Comp(1 + 0.25)	0.02	0.07	0.09	4
Comp(1 + 0.5)		0.14	0.16	2
Comp(1 + 1)		0.28	0.30	1
Comp(1 + 2)		0.56	0.58	0.5

**Table 2:** Charge density of Chit, PNIPAM and Chit-*g*-PNIPAM.

Sample	Volume Titrant (mL) (Method 1)	Volume Titrant (mL) (Method 2)	meq/g	Initially Uncharged Functional Groups (%)
Chit	1.89	1.88	1.65	33.58
PNIPAM	0.17	-	0.15	17.34
Chit-*g*-PNIPAM	2.95	3.03	2.18	39.47

## Data Availability

Data presented in this study are available on reasonable request from the corresponding authors.
